# Factors Associated with Early Discharge after Non-Emergent Right Colectomy for Colon Cancer: A NSQIP Analysis

**DOI:** 10.3390/curroncol30020189

**Published:** 2023-02-18

**Authors:** Malcolm H. Squires, Erin E. Donahue, Michelle L. Wallander, Sally J. Trufan, Reilly E. Shea, Nicole F. Lindholm, Joshua S. Hill, Jonathan C. Salo

**Affiliations:** 1 Division of Surgical Oncology, Levine Cancer Institute, Atrium Health, Charlotte, NC 28204, USA; 2 Department of Biostatistics, Levine Cancer Institute, Atrium Health, Charlotte, NC 28204, USA; 3 Clinical Trials Office, Levine Cancer Institute, Atrium Health, Charlotte, NC 28204, USA

**Keywords:** early discharge, length of stay, colectomy, colon cancer, NSQIP

## Abstract

The National Surgical Quality Improvement Project (NSQIP) dataset was used to identify perioperative variables associated with the length of stay (LOS) and early discharge among cancer patients undergoing colectomy. Patients who underwent non-emergent right colectomy for colon cancer from 2012 to 2019 were identified from the NSQIP and colectomy-targeted databases. Postoperative LOS was analyzed based on postoperative day (POD) of discharge, with patients grouped into Early Discharge (POD 0–2), Standard Discharge (POD 3–5), or Late Discharge (POD ≥ 6) cohorts. Multivariable ordinal logistic regression was performed to identify risk factors associated with early discharge. The NSQIP query yielded 26,072 patients: 3684 (14%) in the Early Discharge, 13,414 (52%) in the Standard Discharge, and 8974 (34%) in the Late Discharge cohorts. The median LOS was 4.0 days (IQR: 3.0–7.0). Thirty-day readmission rates were 7% for Early Discharge, 8% for Standard Discharge, and 12% for Late Discharge. On multivariable regression analysis, risk factors significantly associated with a shorter LOS included independent functional status, minimally invasive approach, and absence of ostomy or additional bowel resection (all *p* < 0.001). Perioperative variables can be used to develop a model to identify patients eligible for early discharge after right colectomy for colon cancer. Efforts to decrease the overall median length of stay should focus on optimization of modifiable risk factors.

## 1. Introduction

The postoperative length of stay (LOS) has become an increasingly scrutinized metric for both surgeons and administrators. Widespread implementation of enhanced recovery after surgery (ERAS) perioperative protocols, particularly among colorectal surgery patients, have helped decrease the postoperative LOS over the last two decades [1,2,3,4,5]. The adoption of minimally invasive surgical approaches, early postoperative mobilization, and other ERAS principles have been associated with decreased postoperative complications and a reduced LOS. Among colorectal surgery patients, the historical published median LOS of 6–7 days has been reduced to a median of 4 days in more recent cohorts and large series [6,7,8]. Several smaller series have noted the feasibility of even earlier discharge after colorectal procedures, with LOS targets of 24–72 h being successfully achieved [9,10,11]. Our own institutional review of patients undergoing elective right colectomy demonstrated that approximately 30% may be clinically ready for discharge on postoperative day one (POD 1) [12]. Other studies, however, have reported an increased risk of postoperative readmission among colorectal cancer patients discharged before POD 4 [13], and efforts to identify patient populations amenable to safe discharge within 48 h post-colectomy have not been widely adopted. Perioperative factors among colorectal cancer patients that may be predictive of successful early discharge have not been well described.

In addition, previous studies have suggested that additional procedures performed during elective colon surgery are associated with an increased risk of postoperative complications [14]; one would hypothesize that additional procedures would similarly be associated with a longer postoperative LOS. The complexity of concurrent procedures has been difficult to quantify; thus, we also sought to create a methodology that would account for all additional procedures in the creation of multivariate models. We sought to characterize postoperative length of stay and readmission risk among patients undergoing colectomy for a right-sided colon cancer within a large, modern cohort, and to analyze perioperative clinical factors associated with early discharge.

## 2. Materials and Methods

Institutional Review Board approval was confirmed prior to initiation of this study (Atrium Health IRB #02-20-31E). The American College of Surgeons National Surgical Quality Improvement Project (NSQIP) [15] general participant use data file (PUF) and colectomy-targeted PUF for 2012–2019 were merged using case ID and subsequently queried by the current procedural terminology (CPT) codes 44205 and 44160 for patients who underwent non-emergent right colectomy for a diagnosis of colon cancer. Emergency cases, cases with missing postoperative LOS, cases with missing operative variables, and cases with an endoscopic approach were excluded.

The postoperative LOS was analyzed as a categorical variable, with patients divided into cohorts designated as Early Discharge (discharge on POD 0–2), Standard Discharge (discharge on POD 3–5), or Late Discharge (discharge on POD ≥ 6). The Discharge cohorts were compared across demographic (age, sex, and race), clinical, and operative characteristics, using chi-square and *t*-tests for categorical and continuous variables, respectively. Clinical characteristics included comorbidities (diabetes, dyspnea, severe chronic obstructive pulmonary disease, congestive heart failure, hypertension, and bleeding disorders), ascites, pre-operative dialysis, pre-operative weight loss, pre-operative sepsis, steroid use, functional status prior to surgery, and tumor stage. Operative characteristics included surgical approach and wound class, and perioperative variables included 30-day readmission and post-operative complications such as wound infection, anastomotic leak (all grades), deep incisional surgical site infection, organ space surgical site infection, pneumonia, urinary tract infection, and deep vein thrombosis.

The NSQIP database captures CPT codes for the primary procedure (colectomy) and up to 10 “Other Procedures” and 10 “Concurrent Procedures” performed at the time of the index operation. We analyzed the nine most common and/or complex additional or concurrent operative procedures as discrete variables: ureteral stent placement, enterolysis, ileostomy, additional bowel resection, cholecystectomy, peritoneal abscess drainage, hysterectomy, ureterolysis, and hepatectomy. The relative value unit (RVU) is a metric to reflect the time and complexity of specific surgical procedures, as designated by the procedural CPT codes. To better analyze the potential complexity of all additional procedures performed at the time of colectomy, we created a new variable termed the “sum RVU” to represent the sum of the RVUs corresponding to CPT codes for all additional or concurrent procedures performed at the time of colectomy, excluding the above nine procedures. The complexity of a surgical case compromising multiple additional procedures in addition to the right colectomy should be reflected in the RVU sum for that case.

To identify risk factors associated with Early Discharge, a multivariable ordinal logistic regression model was fit by including individually prognostic variables and then by using backwards elimination. Statistical analyses were performed using SAS software (SAS Institute, Cary, NC, USA), with *p*-values < 0.05 considered statistically significant.

## 3. Results

**Figure 1:**
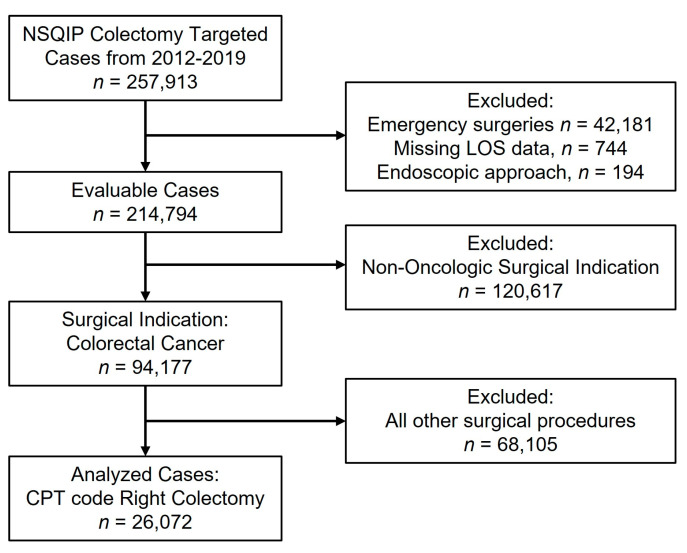
Consort diagram. LOS, length of stay; CPT, current procedural terminology.

A total of 26,072 patients who underwent non-emergent right colectomy for a diagnosis of colon cancer from 2012 to 2019 were identified from the NSQIP query (Figure 1). The median LOS was 4.0 days (interquartile range, IQR: 3.0–7.0) for all patients. The study population comprised 3684 patients (14%) within the Early Discharge (POD 0–2) cohort, 13,414 patients (52%) within the Standard Discharge (POD 3–5) cohort, and 8974 patients (34%) within the Late Discharge (POD ≥ 6) cohort. Thirty-day readmission rates were 7% for the Early Discharge cohort, 8% for the Standard Discharge cohort, and 12% for the Late Discharge cohort.

The clinicopathologic features of the patients across these three cohorts are summarized in Table 1. There was a clear trend towards increasing rates of Early Discharge with each subsequent year, over the course of the 8-year period (2.8% in 2012 vs. 20.2% in 2019). Patients within the Early Discharge cohort were less likely to have diabetes, tobacco use, chronic obstructive pulmonary disease, ascites, congestive heart failure, hypertension, end stage renal disease requiring dialysis, disseminated cancer, non-independent preoperative functional status, preoperative wound infection, chronic steroid use, >10% body weight loss in prior 6 months, bleeding disorders, or a preoperative diagnosis of sepsis. Patients in the Early Discharge cohort were less likely to undergo any additional procedures at the time of colectomy and less likely to require urgent surgery. Patients undergoing minimally invasive surgery (MIS) or MIS with open assist were more likely to fall within the Early Discharge cohort than those undergoing open colectomy or MIS converted to open resection.

**Figure 2:**
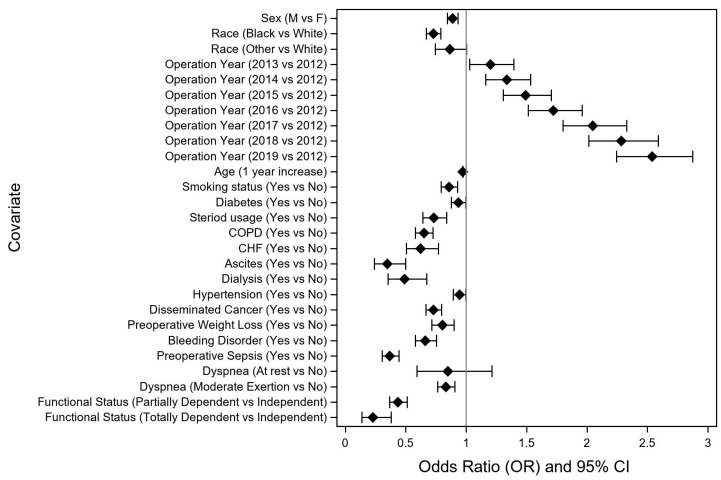
Impact of preoperative variables on likelihood of early discharge (odds ratio > 1) after non-emergent right colectomy for colon cancer. COPD, chronic obstructive pulmonary disease; CHF, congestive heart failure.

**Figure 3:**
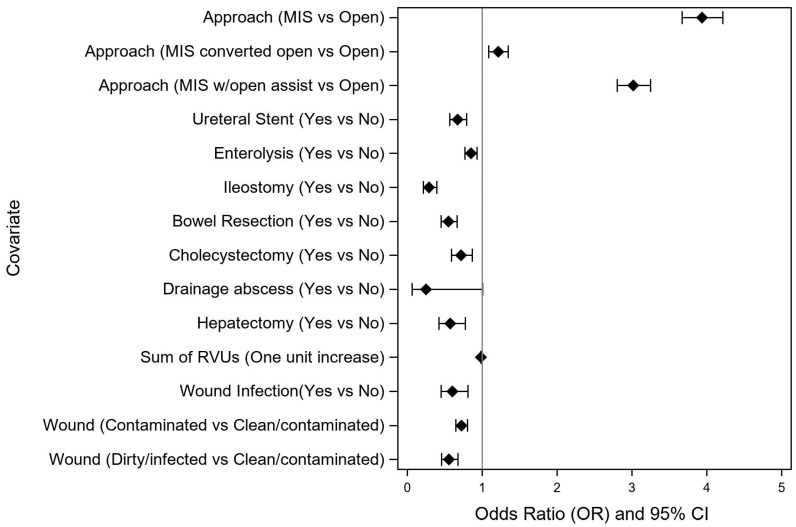
Impact of intraoperative factors on likelihood of early discharge (odds ratio > 1) after non-emergent right colectomy for colon cancer. MIS, minimally-invasive surgery; RVU, relative value unit.

The results from the multivariable ordinal logistic regression model, demonstrating the odds of a shorter length of stay, are included in Table 2. Of the significant univariate factors, ureterolysis, total abdominal hysterectomy (TAH), and body mass index (BMI) were not significant after backwards elimination in the final multivariable model and thus do not appear in Table 2. The impact of preoperative variables on early discharge is shown in Figure 2, while the impact of intraoperative and postoperative variables is shown in Figure 3.

## 4. Discussion

Using the NSQIP dataset, we aimed to identify perioperative variables associated with LOS and early discharge among patients undergoing colectomy for cancer. The indication for surgery has been shown to be correlated with postoperative LOS, with the suggestion that colon cancer diagnosis was negatively associated with Early Discharge [11]. Our institutional experience, however, had suggested that colon cancer diagnosis was not an obvious independent predictor of longer LOS and that up to a third of these patients might be safely discharged by POD 2 [12]. By limiting the current NSQIP analysis to patients with a diagnosis of colon cancer, we sought to eliminate the potential confounding impact of non-malignancy diagnoses. In addition, we elected to focus our analysis on patients undergoing right colectomy for colon cancer to create a more homogenous cohort and minimize some of the confounding operative variables that would have been introduced by including patients undergoing resection of left-sided colon cancers or rectal cancers.

The analysis focused on perioperative clinicopathologic variables associated with postoperative LOS and Early Discharge, using the definition of <48 h. Out of 26,072 patients in the entire study, 3684 (14%) were within the Early Discharge cohort, 13,414 (52%) were within the Standard Discharge cohort, and 8974 (34%) were within the Late Discharge cohort. Importantly, the 30-day postoperative readmission rate was in fact the lowest among the Early Discharge cohort (7% Early Discharge vs. 8% Standard Discharge vs. 12% Late Discharge), suggesting that efforts to appropriately and safely reduce the LOS after colectomy have not led to increased risk of readmission. Previous studies have largely focused on the interaction of postoperative complications, length of stay, and readmission risk; predictably, postoperative complications were found to be associated with an increased postoperative length of stay and decreased likelihood of early discharge [8,11,16,17]. As a clinical goal of the current study was to assist the clinician in identifying which patients might be the best candidates for successful discharge within 48 h after colectomy, we did not include postoperative complications in the multivariable analysis, as these obviously can impact overall LOS but almost invariably would occur after POD 2, the primary endpoint.

Among intraoperative variables, the surgical approach was the single most impactful variable, with patients undergoing MIS colectomy associated with a nearly four-fold increased likelihood of Early Discharge compared to those undergoing an open approach. These data also demonstrated that MIS with open assist was still associated with significantly greater odds of Early Discharge than the open approach or MIS converted to the open approach, suggesting that, when appropriate, the use of a hand port to facilitate completion of safe resection may be a much more beneficial intermediate step than conversion to laparotomy. Not surprisingly, the need for additional bowel resection or ostomy creation were negatively associated with likelihood of Early Discharge. The novel RVU metric we calculated to account for the complexity of additional procedures at the time of colectomy may also serve as a useful tool for discharge predictions and planning. In fact, the mean sum RVUs was the lowest for the Early Discharge cohort and the highest for the Late Discharge cohort.

Among preoperative variables, the presence of ascites, preoperative end stage renal disease with dialysis, preoperative sepsis, and non-independent functional status had the largest negative effect on the likelihood of Early Discharge. By comparison, the negative associations of age, diabetes, smoking status, hypertension, and dyspnea with Early Discharge were modest. There was a modest association of race with the discharge cohort, with Black patients less likely to fall in the Early Discharge cohort. A striking temporal trend was observed, with the proportion of Early Discharge patients steadily increasing over the 8-year study period, from 2.8% in 2012 to 20.2% in 2019. This phenomenon is likely partially attributable to increasing adoption of ERAS protocols across institutions, although these data are not captured within the NSQIP data set. The impact of year of surgery on the likelihood of Early Discharge was notably independent of the surgical approach on multivariable regression analysis, suggesting that improving perioperative management of colectomy patients is associated with the increased likelihood of Early Discharge, independent of the increasing adoption of MIS approaches for colorectal cancer. Interestingly, the proportion of patients in the Standard Discharge cohort (POD 3–5) did not change at all over the study period (51.7% in 2012 vs. 51.6% in 2019), suggesting that there is likely a significant subset of patients within this cohort who could be targeted and would be amenable to safe earlier discharge.

Limitations of the current study include the retrospective nature of the analysis, and while the NSQIP data set is quite comprehensive, data are limited to those derived from participating hospitals. In addition, specific perioperative variables, for example details of ERAS pathway components, are not included. Machine learning and predictive analytics may offer another avenue for the development of postoperative discharge and hospital readmission risk models, although few of these studies to date have included postoperative patients [16,18,19]. A recent study by Xue et al. utilized machine learning algorithms to predict five common postoperative complications based on preoperative and intraoperative data [20]. Predictive modeling using such granular data at an institutional level may offer additional opportunities to shape personalized postoperative care pathways for patients undergoing colectomy in the future [21] but NSQIP provides a unique, high-quality dataset to currently study postoperative outcomes at a national level.

The decision-making process regarding when to discharge a postoperative patient after surgery is a complex one that requires skilled clinical judgement. The current study provides insights from a large, modern cohort by using the NSQIP database to examine postoperative length of stay and the perioperative clinical factors associated with early discharge among patients undergoing colectomy for right-sided colon cancers. Risk factors significantly associated with early discharge on multivariable analysis included independent functional status, minimally invasive approach, and the absence of ostomy or additional bowel resection. Importantly, Early Discharge was not associated with any increase in readmission, suggesting that early recovery strategies as currently practiced do not represent a risk to patient safety. The use of RVU-based metrics to quantify surgical complexity further aids in the discharge decision-making process by confirming that patients undergoing less complex operations are more likely to qualify for Early Discharge. Together, these observations provide valuable information for clinical decision-making in surgical patients. Efforts to safely decrease the median length of stay and improve resource utilization after non-emergent colectomy should focus on the optimization of modifiable risk factors and the identification of patient populations from this model in which early discharge could be safely achieved.

## Figures and Tables

**Table 1:** Clinical and demographic factors of patients undergoing non-emergent right colectomy for colon cancer from 2012 to 2019, stratified by postoperative length of stay.

Factor		Total	Early	Standard	Late
		*N* = 26,072	Discharge	Discharge	Discharge
			*N* = 3684	*N* = 13,414	*N* = 8974
Age, mean (SD), years		69.1 (12.9)	65.3 (11.9)	68.3 (12.8)	71.8 (12.8)
Sex	Female	13,874 (53.2%)	1899 (13.7%)	7422 (53.5%)	4553 (32.8%)
	Male	12,198 (46.8%)	1785 (14.6%)	5992 (49.1%)	4421 (36.2%)
Race	Black	2839 (10.9%)	359 (12.6%)	1421 (50.1%)	1059 (37.3%)
	Other	750 (2.9%)	119 (15.9%)	396 (52.8%)	235 (31.3%)
	Unknown	4306 (16.5%)	487 (11.3%)	2226 (51.7%)	1593 (37.0%)
	White	18,177 (69.7%)	2719 (15.0%)	9371 (51.6%)	6087 (33.5%)
Current Smoker	No	22,895 (87.8%)	3270 (14.3%)	11,832 (51.7%)	7793 (34.0%)
	Yes	3177 (12.2%)	414 (13.0%)	1582 (49.8%)	1181 (37.2%)
Diabetes	No	20,753 (79.6%)	3079 (14.8%)	10,663 (51.4%)	7011 (33.8%)
	Yes	5319 (20.4%)	605 (11.4%)	2751 (51.7%)	1963 (36.9%)
Dyspnea	No	23,188 (88.9%)	3434 (14.8%)	12,058 (52.0%)	7696 (33.2%)
	At Rest	142 (0.5%)	9 (6.3%)	58 (40.8%)	75 (52.8%)
	Moderate Exertion	2742 (10.5%)	241 (8.8%)	1298 (47.3%)	1203 (43.9%)
History of Severe COPD	No	24,474 (93.9%)	3572 (14.6%)	12,718 (52.0%)	8184 (33.4%)
	Yes	1598 (6.1%)	112 (7.0%)	696 (43.6%)	790 (49.4%)
CHF (30 Days Before Surgery)	No	25,647 (98.4%)	3659 (14.3%)	13,254 (51.7%)	8734 (34.1%)
	Yes	425 (1.6%)	25 (5.9%)	160 (37.6%)	240 (56.5%)
Hypertension Requiring Medication	No	10,957 (42.0%)	1767 (16.1%)	5825 (53.2%)	3365 (30.7%)
	Yes	15,115 (58.0%)	1917 (12.7%)	7589 (50.2%)	5609 (37.1%)
Ascites	No	25,889 (99.3%)	3680 (14.2%)	13,372 (51.7%)	8837 (34.1%)
	Yes	183 (0.7%)	4 (2.2%)	42 (23.0%)	137 (74.9%)
Currently on Dialysis (Pre-operative)	No	25,895 (99.3%)	3674 (14.2%)	13,343 (51.5%)	8878 (34.3%)
	Yes	177 (0.7%)	10 (5.6%)	71 (40.1%)	96 (54.2%)
T Stage	T0 or Tis	530 (2.0%)	118 (22.3%)	292 (55.1%)	120 (22.6%)
	T1	2388 (9.2%)	446 (18.7%)	1339 (56.1%)	603 (25.3%)
	T2	3809 (14.6%)	659 (17.3%)	2065 (54.2%)	1085 (28.5%)
	T3	12,360 (47.4%)	1669 (13.5%)	6437 (52.1%)	4254 (34.4%)
	T4	4728 (18.1%)	430 (9.1%)	2180 (46.1%)	2118 (44.8%)
	Tx, N/A, Unknown	2257 (8.7%)	362 (16.0%)	1101 (48.8%)	794 (35.2%)
N Stage	N0	13,795 (52.9%)	2071 (15.0%)	7257 (52.6%)	4467 (32.4%)
	N1	6454 (24.8%)	857 (13.3%)	3314 (51.3%)	2283 (35.4%)
	N2	3472 (13.3%)	385 (11.1%)	1705 (49.1%)	1382 (39.8%)
	Nx, N/A, Unknown	2351 (9.0%)	371 (15.8%)	1138 (48.4%)	842 (35.8%)
M Stage	M0 or Mx	13,313 (51.1%)	2085 (15.7%)	6909 (51.9%)	4319 (32.4%)
	M1	1780 (6.8%)	119 (6.7%)	781 (43.9%)	880 (49.4%)
	N/A, Unknown	10,979 (42.1%)	1480 (13.5%)	5724 (52.1%)	3775 (34.4%)
Disseminated Cancer	No	23,397 (89.7%)	3495 (14.9%)	12,266 (52.4%)	7636 (32.6%)
	Yes	2675 (10.3%)	189 (7.1%)	1148 (42.9%)	1338 (50.0%)
Pre-Operative Weight Loss (>10% in Last 6 Months)	No	24,618 (94.4%)	3581 (14.5%)	12,793 (52.0%)	8244 (33.5%)
	Yes	1454 (5.6%)	103 (7.1%)	621 (42.7%)	730 (50.2%)
Bleeding Disorders	No	25,030 (96.0%)	3617 (14.5%)	12,969 (51.8%)	8444 (33.7%)
	Yes	1042 (4.0%)	67 (6.4%)	445 (42.7%)	530 (50.9%)
Pre-Operative Sepsis	No	25,435 (97.6%)	3672 (14.4%)	13,241 (52.1%)	8522 (33.5%)
	Yes	637 (2.4%)	12 (1.9%)	173 (27.2%)	452 (71.0%)
Steroid Use for Chronic Conditions	No	25,084 (96.2%)	3579 (14.3%)	12,960 (51.7%)	8545 (34.1%)
	Yes	988 (3.8%)	105 (10.6%)	454 (46.0%)	429 (43.4%)
Functional Health	Independent	25,166 (96.5%)	3647 (14.5%)	13,115 (52.1%)	8404 (33.4%)
Status Prior to Surgery	Partially Dependent	719 (2.8%)	29 (4.0%)	230 (32.0%)	460 (64.0%)
	Totally Dependent	91 (0.3%)	0 (0.0%)	25 (27.5%)	66 (72.5%)
	Unknown	96 (0.4%)	8 (8.3%)	44 (45.8%)	44 (45.8%)
Year of Operation	2012	1411 (5.4%)	40 (2.8%)	729 (51.7%)	642 (45.5%)
	2013	1530 (5.9%)	68 (4.4%)	810 (52.9%)	652 (42.6%)
	2014	2357 (9.0%)	184 (7.8%)	1233 (52.3%)	940 (39.9%)
	2015	3066 (11.8%)	313 (10.2%)	1565 (51.0%)	1188 (38.7%)
	2016	3716 (14.3%)	497 (13.4%)	1893 (50.9%)	1326 (35.7%)
	2017	3993 (15.3%)	629 (15.8%)	2077 (52.0%)	1287 (32.2%)
	2018	4581 (17.6%)	858 (18.7%)	2314 (50.5%)	1409 (30.8%)
	2019	5418 (20.8%)	1095 (20.2%)	2793 (51.6%)	1530 (28.2%)
Surgery Approach	MIS	10,645 (40.8%)	2270 (21.3%)	6040 (56.7%)	2335 (21.9%)
	MIS Converted Open	1883 (7.2%)	80 (4.2%)	833 (44.2%)	970 (51.5%)
	MIS with Open	6907 (26.5%)	1167 (16.9%)	3948 (57.2%)	1792 (25.9%)
	Assist				
	Open	6637 (25.5%)	167 (2.5%)	2593 (39.1%)	3877 (58.4%)
Any Additional Procedure	No	16,898 (64.8%)	2824 (16.7%)	9169 (54.3%)	4905 (29.0%)
	Yes	9174 (35.2%)	860 (9.4%)	4245 (46.3%)	4069 (44.4%)
Urgent Surgery	No	21,138 (81.1%)	3489 (16.5%)	11,579 (54.8%)	6070 (28.7%)
	Yes	4934 (18.9%)	195 (4.0%)	1835 (37.2%)	2904 (58.9%)
Ureteral Stent	No	25,436 (97.6%)	3655 (14.4%)	13,125 (51.6%)	8656 (34.0%)
	Yes	636 (2.4%)	29 (4.6%)	289 (45.4%)	318 (50.0%)
Enterolysis	No	24,110 (92.5%)	3488 (14.5%)	12,518 (51.9%)	8104 (33.6%)
	Yes	1962 (7.5%)	196 (10.0%)	896 (45.7%)	870 (44.3%)
Ileostomy	No	25,787 (98.9%)	3676 (14.3%)	13,357 (51.8%)	8754 (33.9%)
	Yes	285 (1.1%)	8 (2.8%)	57 (20.0%)	220 (77.2%)
Bowel Resection	No	25,535 (97.9%)	3670 (14.4%)	13,231 (51.8%)	8634 (33.8%)
	Yes	537 (2.1%)	14 (2.6%)	183 (34.1%)	340 (63.3%)
Cholecystectomy	No	25,611 (98.2%)	3652 (14.3%)	13,212 (51.6%)	8747 (34.2%)
	Yes	461 (1.8%)	32 (6.9%)	202 (43.8%)	227 (49.2%)
Abscess Requiring	No	26,050 (99.9%)	3684 (14.1%)	13,411 (51.5%)	8955 (34.4%)
Drainage	Yes	22 (0.1%)	0 (0.0%)	3 (13.6%)	19 (86.4%)
Total Abdominal Hysterectomy	No	25,983 (99.7%)	3679 (14.2%)	13,375 (51.5%)	8929 (34.4%)
	Yes	89 (0.3%)	5 (5.6%)	39 (43.8%)	45 (50.6%)
Ureterolysis	No	26,028 (99.8%)	3682 (14.1%)	13,398 (51.5%)	8948 (34.4%)
	Yes	44 (0.2%)	2 (4.5%)	16 (36.4%)	26 (59.1%)
Hepatectomy	No	25,861 (99.2%)	3678 (14.2%)	13,344 (51.6%)	8839 (34.2%)
	Yes	211 (0.8%)	6 (2.8%)	70 (33.2%)	135 (64.0%)
Sum RVUs Remaining Additional Procedures, Mean (SD)		4.0 (11.0)	1.6 (5.2)	2.9 (8.2)	6.6 (15.2)
Wound Infection	No	25,836 (99.1%)	3677 (14.2%)	13,333 (51.6%)	8826 (34.2%)
	Yes	236 (0.9%)	7 (3.0%)	81 (34.3%)	148 (62.7%)
Wound Class	Clean/	23,928 (91.8%)	3520 (14.7%)	12,563 (52.5%)	7845 (32.8%)
	Contaminated				
	Contaminated	1575 (6.0%)	138 (8.8%)	687 (43.6%)	750 (47.6%)
	Dirty/Infected	569 (2.2%)	26 (4.6%)	164 (28.8%)	379 (66.6%)
Anastomotic Leak	No	25,373 (97.3%)	3637 (14.3%)	13,229 (52.1%)	8507 (33.5%)
	Yes	643 (2.5%)	43 (6.7%)	153 (23.8%)	447 (69.5%)
	Unknown	56 (0.2%)	4 (7.1%)	32 (57.1%)	20 (35.7%)
Deep Incisional Surgical Site Infection	No	25,924 (99.4%)	3675 (14.2%)	13,377 (51.6%)	8872 (34.2%)
	Yes	148 (0.6%)	9 (6.1%)	37 (25.0%)	102 (68.9%)
Organ Space Surgical Site Infection	No	25,159 (96.5%)	3635 (14.4%)	13,193 (52.4%)	8331 (33.1%)
	Yes	913 (3.5%)	49 (5.4%)	221 (24.2%)	643 (70.4%)
Pneumonia	No	25,543 (98.0%)	3676 (14.4%)	13,352 (52.3%)	8515 (33.3%)
	Yes	529 (2.0%)	8 (1.5%)	62 (11.7%)	459 (86.8%)
Urinary Tract Infection	No	25,639 (98.3%)	3662 (14.3%)	13,284 (51.8%)	8693 (33.9%)
	Yes	433 (1.7%)	22 (5.1%)	130 (30.0%)	281 (64.9%)
DVT/ Thrombophlebitis	No	25,744 (98.7%)	3668 (14.2%)	13,317 (51.7%)	8759 (34.0%)
	Yes	328 (1.3%)	16 (4.9%)	97 (29.6%)	215 (65.5%)

The Early Discharge cohort included patients discharged on postoperative day 0–2, the Standard Discharge cohort included patients discharged on postoperative day 3–5, and the Late Discharge cohort included patients discharged on postoperative day ≥6. COPD, chronic obstructive pulmonary disease; CHF, congestive heart failure; DVT, deep vein thrombosis; MIS, minimally invasive surgery; RVU, relative value unit; SD, standard deviation.

**Table 2:** Multivariable ordinal logistic regression model of risk factors associated with Early Discharge (POD 0–2).

Factor		Odds Ratio	95% CI	*p* Value
Age, 1-year increase		0.972	0.970, 0.974	<0.0001
Sex	Male vs. Female	0.887	0.843, 0.932	<0.0001
Race	Black vs. White	0.728	0.671, 0.791	<0.0001
	Other vs. White	0.864	0.746, 1.002	0.0534
	Unknown/Not Reported vs. White	0.664	0.620, 0.712	<0.0001
Smoking status	Yes vs. No	0.859	0.794, 0.928	0.0001
Diabetes	Yes vs. No	0.934	0.877, 0.996	0.0367
Dyspnea	At Rest vs. No	0.847	0.592, 1.213	0.3661
	Moderate Exertion vs. No	0.831	0.763, 0.906	<0.0001
History of Severe COPD	Yes vs. No	0.650	0.581, 0.727	<0.0001
CHF (30 Days Before Surgery)	Yes vs. No	0.623	0.505, 0.769	<0.0001
Ascites	Yes vs. No	0.346	0.239, 0.499	<0.0001
Currently on Dialysis (Pre-Operative)	Yes vs. No	0.489	0.355, 0.673	<0.0001
Disseminated Cancer	Yes vs. No	0.729	0.667, 0.797	<0.0001
Pre-Operative Weight Loss (>10% in Last 6 Months)	Yes vs. No	0.802	0.716, 0.898	0.0001
Bleeding Disorder	Yes vs. No	0.661	0.580, 0.754	<0.0001
Pre-Operative Sepsis	Yes vs. No	0.368	0.304, 0.446	<0.0001
Steroid	Yes vs. No	0.733	0.643, 0.837	<0.0001
Functional Status	Partially Dependent vs. Independent	0.434	0.366, 0.513	<0.0001
	Totally Dependent vs. Independent	0.228	0.136, 0.380	<0.0001
	Unknown vs. Independent	0.761	0.502, 1.153	0.1973
Year of Operation	2013 vs. 2012	1.199	1.031, 1.396	0.0188
	2014 vs. 2012	1.335	1.162, 1.533	<0.0001
	2015 vs. 2012	1.493	1.308, 1.704	<0.0001
	2016 vs. 2012	1.722	1.513, 1.958	<0.0001
	2017 vs. 2012	2.048	1.802, 2.327	<0.0001
	2018 vs. 2012	2.283	2.013, 2.589	<0.0001
	2019 vs. 2012	2.538	2.242, 2.873	<0.0001
Surgery Approach	MIS vs. Open	3.934	3.672, 4.214	<0.0001
	MIS converted open vs. Open	1.209	1.087, 1.345	0.0005
	MIS with open assist vs. Open	3.016	2.802, 3.247	<0.0001
Ureteral Stent	Yes vs. No	0.669	0.566, 0.791	<0.0001
Enterolysis	Yes vs. No	0.845	0.768, 0.930	0.0006
Ileostomy	Yes vs. No	0.286	0.211, 0.388	<0.0001
Bowel Resection	Yes vs. No	0.545	0.449, 0.661	<0.0001
Cholecystectomy	Yes vs No	0.714	0.587, 0.867	0.0007
Drainage Abscess	Yes vs. No	0.247	0.061, 1.005	0.0509
Hepatectomy	Yes vs. No	0.569	0.417, 0.775	0.0004
Sum Remaining RVUs additional procedures (one unit increase)		0.982	0.979, 0.985	<0.0001
Wound Infection	Yes vs. No	0.601	0.448, 0.808	0.0007
Wound Class	Contaminated vs. Clean/Contaminated	0.719	0.646, 0.800	<0.0001
	Dirty/Infected vs. Clean/Contaminated	0.553	0.454, 0.674	<0.0001

## Data Availability

The data presented in this study are available on request from the corresponding author.
